# The Tooth in the Apple

**Published:** 1889-04

**Authors:** 


### The Tooth in the Apple.

Chandler Jones, a negro, is in jail for a burglary on Mr. Milton's store in Hazelhurst. The circumstances of his detection are peculiar, and the work was done by Detective E. A. Wilson, who had found nothing in the way of a clue except an apple, out of which two bites had been taken. He at once noticed that the two front teeth of the biter were not only irregular, but peculiar. He imagined that when the biter was a boy an old tooth remainining in the gum caused a new tooth to grow one-sided. The apple was placed in water so as to prevent shriveling, and keeping his secret to himself, Wilson went down to Baxley, where he knew a number of loafing negroes.

Walking into a store he bought some apples and biting one, said to a well-dressed negro who had attracted his attention: "Try one." The negro accepted the gift, and when he raised the apple to his mouth for a second bite the handcuffs were placed on his wrists. There never was a more astonished negro. He was under arrest so quickly that he was unable to offer any resistance. He gave his name as Chandler Jones, and was found to be wearing a suit of clothes and a watch and chain taken from Mr. Milton. Jones was taken to the store, where he showed how he obtained entrance on the night of the burglary, and how the first thing he saw was a barrel of apples. He picked up one, and after two bites laid it down on Mr. Milton's desk.—*Macon Telegraph*, (*Ga*).

### To the Editor of The Dental Register.

DEAR SIR:—At the regular meeting of the Chicago Dental Society, held Tuesday evening, March 5, 1889, the following resolution was adopted:

*Rosolved*, That it is the sentiment of this Society, that it would be of interest to the members of dental profession to become members of the Dental Protective Association of the United States.

LOUIS OTTOFY,
Corresponding Secretary.

THE most heavily endowed educational institutions in the United States are Girard College, $10,000,000; Columbia, $5,-000,000; Johns Hopkins, $4,000,000; Princeton, $3,500,000, and Harvard, $3,000,000.

MRS. PALID—"Don't you find that the noise of the boiler foundry across the street affects your nerves, Mrs. Youngwife?"

Mrs. Youngwife—"I seldom hear it."

"How strange!"

"Well, you see, baby is teething now."—*Drake's Magazine.*

IODIZED GLYCERINE.—Dr. G. Hammond points out that a mixture of tincture of iodine and glycerine produces a greater effect on the skin than the pure tincture, possibly because the glycerine tends to prevent the evaporation of the iodine, and thus enables the whole of its powers to be utilized.—*Med. Record.*

SURGEON-GENERAL John B. Hamilton announces that in view of the recent enactment of a law prohibiting any original appointments in the Marine Hospital Service, except to the rank of Assistant-Surgeon, thereby practically creating a life-tenure in the office of Supervising Surgeon-General, he has resigned the editorship of the *Journal of the American Medical Association.*

TOBACCO ON THE EYES.—The eyes of some are especially susceptible to tobacco. Those who feel the slightest nausea or giddiness after using tobacco should let it alone.

There is a woman in this city who smoked only one cigar a day for three years, but who at the end of that time was afflicted with tobacco amaurosis of both eyes; nor has she ever regained her sight.—*Keyser.*

THE use of yeast in therapeutics, while not new, is recommended for scurvy by Heer, who treated 800 scorbutic patients with it, of whom but two died. The mortality of other scorbutic patients (prisoners), not treated with it was much greater. Heer attributes to it some action on the fever and the course of the disease. Under its use the temperature fell from 41° to 38° C. in twelve hours.

FOR WHAT ARE DOCTORS PAID?—An English judge has recently given a decision containing a great deal of common sense. A local surgeon sued the executor of a deceased farmer for an amount for consultations. The deceased could not take any medicine. The judge in giving his decision said: "Many people of a better class hold the idea that it is medicine doctors are paid for. It is for skill."

Danger in Large Doses of Quinine.—Quinine sometimes affects the eyes dangerously. He relates the case of a man who came to him from Ohio, several years ago, totally blind. This man had, in the attempt to doctor himself for an attack of malaria, taken two doses of quinine, an hour apart, each consisting of a heaping tablespoonful of the drug. Blindness followed in a few hours; and treatment proved of no avail; the man's sight is still wanting.—*Keyser.*

Camphorated Naphthol —It has recently been found that naphthol, which is very insoluble in water, can be combined with camphor so as to form a liquid. It is only necessary to rub up one part of naphthol with two parts of dry camphor to obtain a creamy liquid. This product is of a pinkish color if the naphthol is not pure. It has been found to be an excellent antiseptic appliaction to wounds and ulcers, and is said to clean off the false membrane in diphtheria.

The Only Substitute.—Stranger (from Dakota): "I hear you are making mustard plasters by the yard, always ready for use."

Druggist: "Yes, sir. Would you like to try one?"

Stranger: "Not now; but if you'll warrant 'em good and hot you can send me about forty yards by express to Blizzardville, Dakota. Winter is coming on and wool is too high for poor folks.—*Philadelphia Medical Record.*

Experimental Iodoform Poisoning.—Dr. A. V. Koriander, of St. Petersburg, has endeavored to throw some light on the vexed question of the suitability of iodoform for use as an antiseptic by poisoning dogs with it, and examining the morbid appearances post mortem. The iodoform was introduced into the peritoneal cavity in quantities varying from 0.3 to 1.5 gram per kilog. of the animal's weight. The microscopic sections of the organs were stained by hæmotoxylin and lithion-carmine. Nephritis affecting the renal glomeruli was invariably found, and the liver was infiltrated by minute fat granules. These appearances are considered by Dr. Philipovich to be characteristic of iodoform poisoning.—*Lancet*, Jan. 19, 1889.

---

GOOD ADVICE.—A doctor in Liverpool has recently written to the press complaining that a prescription which he gave to a patient was being used by that patient to cure a great number of his friends. He told the man, who was a carpenter, that it was just as unfair to lend his prescription as it would be for the doctor to borrow the jointer's tools and lend them to his friends. He suggested that the proper way for the patient to do was to send these sick people to him. The moral he deduces is, that doctors should not give prescriptions to patients. If the doctor does not dispense, he should send the patient with the prescription, under cover, to a chemist, who should have instructions not to deliver a copy of it to the patient unless specially ordered to do so.—*Canada Medical Record.*

---

DIGESTIBILITY AS A TEST OF FOOD VALUE.—Dr. Sarah E. Post, discussing this question, concludes that:

1. Digestibility is not a complete test of food value.
2. Proteides are of the most value when limited in quantity and of the less easily digested forms.
3. Limitation of proteid food is particularly indicated in Bright's disease.
4. The sphygmograph furnishes important evidence in the pre-albuminuric stage of this condition.

Finally, "predigested foods" and "easily digested foods" should be reserves for cases in which the digestion is in fault. The value of such foods in the management of the pathological conditions of the digestive tract is beyond question.—*Dietetic Gazette.*

---

MORE HOSPITALS THAN MONEY FOR THE SICK.—Dr. Sutherland, who has recently written an interesting series of papers concerning the condition of the voluntary hospitals of Great Britain, gives the following statistics concerning the voluntary hospitals of England, Scotland, and Ireland, not including the infirmaries sustained by the poor-law rates. Dublin has one hospital bed to every 140 of the population; Belfast one to 380; Edinburgh one to 410; Londen one to 420; Birmingham one to 700; Sheffield one to 830, and Leeds one to 1,020. In Glasgow there are 1,057 beds daily occupied in the voluntary hospitals at an annual expense of $220 per bed, while there are 1,169 beds daily occupied in the hospitals supported by the poor-law rates at an annual expense of only $115 per bed. The annual deficit in the working expenses of the voluntary hospitals of the last-named city is stated at $45,000. And the *British Medical Journal*, for February 2, 1889, in stating the foregoing facts, adds that, this impecunious state of the voluntary hospitals in London is still worse.

Dangers from the Use of Cocaine.—The following conclusions are taken from an editorial on this subject in the *Maryland Medical Jonrnal:*

1. Certain persons possess an idiosyncrasy to cocaine which can not be foreseen or entirely guarded against.
2. Cocaine exerts its toxic effects upon the nervous centres and, secondarily the heart.
3. Its evil effects are most liable to be seen in neurotic subjects.
4. The danger in cocaine poisoning is mainly from paralysis of the heart, syncope.
5. It may be well to precede its use by the adminstration of alcohol or other cardiac stimulants as is done with chloroform.
6. Special care is needed in "weak heart" and organic heart disease.
7. The subcutaneous administration is dangerous, and should be avoided.
8. The use of the stronger solutions is dangerous and unnecessary.
9. The treatment of cocaine poisoning consists of measures to rouse the heart, especially of inhalations of nitrite of amyl.

